# Condensed Facts Concerning Electricity for the General Practitioner

**Published:** 1897-04

**Authors:** 


### Selections and Abstracts.

### CONDENSED FACTS CONCERNING ELECTRICITY FOR THE GENERAL PRACTITIONER.

General practitioners desiring to use electricity in their practice, but possessing practically no knowledge of the electro-therapeutics, will do well to read, study, *and learn* the following points:

The faradic or interrupted current cures disease mainly through physiological and mechanical processes, producing no appreciable chemical effect in the tissues.

In each faradic battery there should be two coils—primary and secondary.

The current from the primary coil has greater quantity and produces greater mechanical effects, but lacks penetrating power, and is more useful in superficial troubles, particularly where mechanical efforts are desired.

It is also of service in internal treatments for many vaginal and uterine diseases.

In the primary current there is some difference in polarity, the positive pole producing greater sedation and the negative pole greater stimulation.

The secondary coil produces a current having much greater penetrating power than the primary current.

It will more easily overcome resistance on account of its greater tension, and is to be preferred to the primary current in treating deeply-seated conditions.

There is no appreciable difference in polarity in the current from the secondary coil.

The current from the secondary coil is much more pleasant in its effect, particularly to nervous subjects.

In the direct galvanic current there are no interruptions to the current as in the physiological and mechanical effects.

There is no sensation to the current beyond a feeling of numbness and burning beneath the electrodes.

In general terms, it may be stated that the faradic current is to be preferred in muscular troubles and the galvanic in nervous diseases.

The galvanic current will produce a greater degree of sedation or stimulation.

The galvanic current should be used to stimulate the absorbents, to remove effusions, morbid growths, callosities, tumors, facial blemishes, superfluous hairs, etc. In these conditions the faradic current is practically powerless.

The positive pole of the galvanic current is much more sedative and the negative pole much more irritating and stimulating.

Electrolysis and cataphoresis can only be used with the galvanic current.

To produce the most pronounced sedative effect and allay irritability make stabile applications of the positive pole, galvanic current.

To produce the most marked irritation, use the negative pole of the galvanic battery or reverse the polarity frequently.

Electricity has a decided refreshing and soothing effect upon the entire nervous system, and is of great service in nearly all nervous diseases.

Either faradic or galvanic current will equalize the circulation, increase the flow of blood to the surface and extremities, and improve nutrition and assimilation.—*The Electro Therapeutist.*

